# Case report: Axilla fibroadenoma – An atypical presentation

**DOI:** 10.1016/j.amsu.2022.104295

**Published:** 2022-08-05

**Authors:** Safna Naozer Virji, Lubna Mushtaque Vohra, Syeda Sakina Abidi, Romana Idrees

**Affiliations:** a Department of Surgery, Aga Khan University Hospital, Stadium Road, Karachi, 74800, Pakistan; b Section of Breast Surgery, Department of Surgery, Aga Khan University Hospital, Stadium Road, Karachi, 74800, Pakistan; c Section of Histopathology, Department of Pathology and Laboratory Medicine, Aga Khan University Hospital, Stadium Road, Karachi, 74800, Pakistan

**Keywords:** Fibroadenoma, Axilla, Accessory breast tissue, Case report

## Abstract

**Introduction:**

Fibroadenoma is the most common benign breast tumor among women between 15 and 35 years, however, a fibroadenoma arising from accessory breast tissue is a rare occurrence.

**Case presentation:**

We encountered this uncommon presentation in a 37 years old female with a gradually increasing left axillary lump associated with discomfort. On ultrasound it was a 17.3 mm × 10.6 mm x 17.5 mm well defined solid nodule with internal vascularity, BiRADS IVa lesion. Core biopsy revealed fibroepithelial lesion and the patient electively underwent excisional biopsy. Histopathology confirmed the diagnosis of fibroadenoma, which was completely excised.

**Clinical discussion:**

Approximately half of all breast lumps in women are diagnosed as fibroadenomas, making it the most common benign breast mass. Nonetheless, an axillary mass has several differentials such as lymphadenopathy, lipoma or sebaceous cyst, while a fibroadenoma developed from ectopic breast tissue in the axilla is an unusual condition. Masses in axilla like ectopic breast tissue are clinically significant as they undergo physiological changes like the normal breast tissue like pain and discomfort during pregnancy, lactation and menstruation. This tissue may also undergo pathological changes and may pose a diagnostic challenge.

**Conclusion:**

Axillary lumps pose a diagnostic dilemma and accessory breast tissue related pathologies should be considered.

## Highlights

• Axillary lumps may pose a diagnostic dilemma.
• Accessory breast tissue may undergo pathological changes and present as benign or malignant breast disease.
• Ectopic breast tissue should undergo routine annual screening.

## 1 Introduction

Among breast tumors, fibroadenoma is the most common benign tumor with an incidence of 25%, usually occurring in women between the ages of 15 and 35 years [1]. In Pakistan, studies have reported that 71.3% of breast lumps are diagnosed to be fibroadenomas [2]. Ultrasound is the preferred modality for investigation in this age group and some sonographic features of fibroadenomas include a well-circumscribed, oval appearing, hypoechoic focal mass that displaces the surrounding parenchyma [3,4].

On histopathology, fibroadenomas are characterized by cellular proliferation of the stroma and glands, with a relatively constant ratio of stroma to glands throughout the lesion. It has a uniform, hypovascular stroma, composed of spindle-shaped cells with bland oval to elongated nuclei with the absence of stromal cell pleomorphism. Although rare, in younger women, stromal mitosis may be seen, however it does not indicate malignancy [5].

Fibroadenomas though one of the most common benign breast lesions, it is rarely reported in literature as arising in accessory breast tissue [6]. Axillary breast tissue may occur in 2–6% of women, and this may undergo changes as in the normal breast tissue which include periodic enlargement of an axillary accessory breast, cyclic pain and even a palpable axillary mass [7,8]. These symptoms may pose a source of severe anxiety. We present a case of a woman with a left axillary lump.

This case has been reported in line with the SCARE 2020 Criteria [9].

## 2 Presentation of case

A 37 years old Pakistani female with no prior comorbid, no risk factors, negative family history of breast cancer, mother of three, presented with a left axillary lump since 2018. She had an unremarkable drug or allergy history. She had a prior history of a left breast infected sebaceous cyst for which she had undergone incision and drainage of the lesion in 2015.

**Fig. 1:**
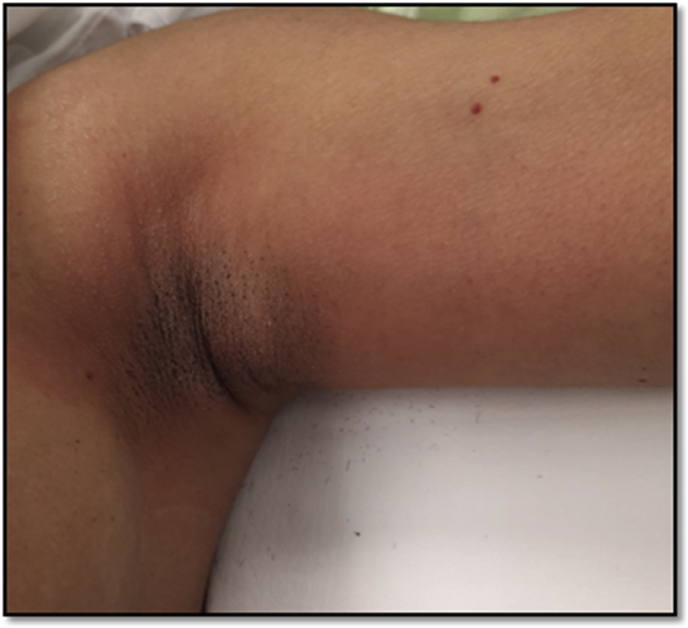
Clinical examination: 2 × 2 cm freely mobile, irregular, firm lump palpable in the left axilla.

**Fig. 2:**
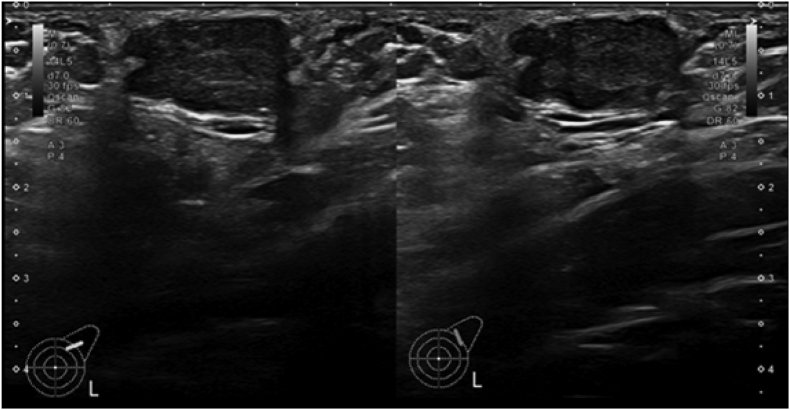
Ultrasound left axilla: 17.3 mm × 10.6 mm x 17.5 mm lobulated superficial hypoechoic well defined solid nodule with internal vascularity.

She presented in March 2022 with the complaint of increase in size of the lump and associated pain. On examination it was a 2 × 2 cm freely mobile irregular firm lump in the left axilla (Fig. 1). Bilateral breast examination was normal. Ultrasound examination of the left breast revealed: Multiple anechoic, thin-walled cysts without internal echoes or solid tissue. The largest cyst measured 39.3 mm × 11 mm at 3:00 o'clock position. No solid lesion was identified in the left breast. The retroareolar region was normal. A mildly lobulated superficial hypoechoic well defined solid nodule with internal vascularity was identified in the left axilla at the site of palpable and visible abnormality measuring 17.3 mm × 10.6 mm x 17.5 mm. BiRADS IVa (Fig. 2). Ultrasound guided core biopsy was performed which revealed a fibroepithelial lesion.

**Fig. 3:**
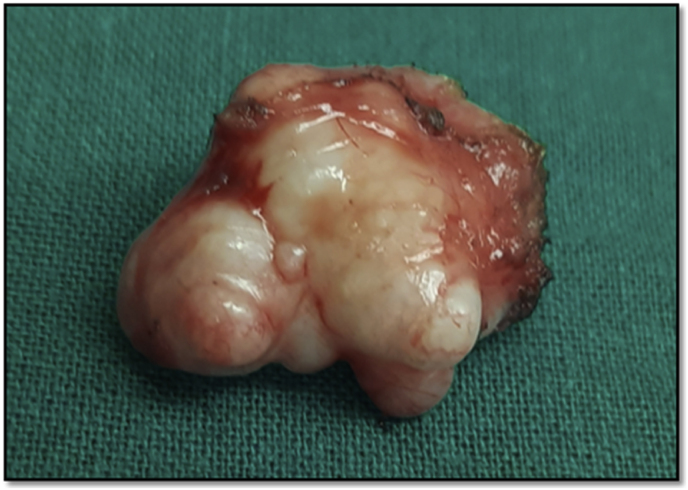
Gross specimen: Fibroadenoma 2.0 × 1.5 × 1.0 cm.

She was counselled and given the option of surveillance with a repeat ultrasound at 6 months, however, the patient elected to undergo early excisional biopsy under general anesthesia by the breast surgeon at a tertiary care hospital. A 2 × 1cm lobulated tumor was excised through an axillary incision and sent for histopathological examination (Fig. 3). Post-procedure, the patient was discharged on the same day..

**Fig. 4:**
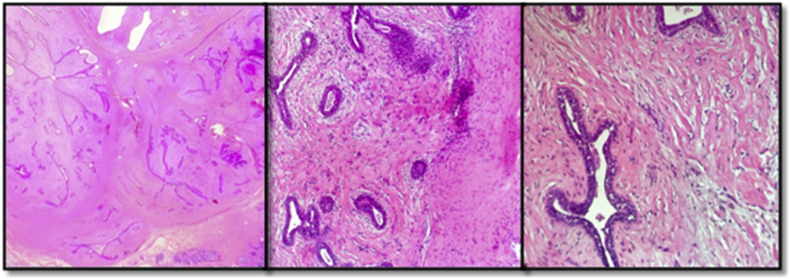
Low and high power magnification showed a well circumscribed biphasic lesion composed of compressed bi-layered ducts surrounded by hypo-cellular stroma. H&E stain.

She followed up in clinic after 10 days. Her incision was healing well and her histopathology revealed a fibroadenoma, which was completely excised (Fig. 4). She suffered no post-operative complications and was advised yearly follow-up, with breast screening starting at the age of 40 years.

## 3 Discussion

In young women, approximately half of all breast lumps are diagnosed as fibroadenomas making it the most common benign breast mass. None the less, an axillary mass has several differentials such as lymphadenopathy, lipoma or sebaceous cyst, while a fibroadenoma developed from ectopic breast tissue in the axilla is an unusual condition [10]. A fibroadenoma typically has the clinical features of a painless, mobile lump that is usually identified on palpation. Radiologically, ultrasound is the preferred tool used to characterize axillary masses; however, for definitive diagnosis histopathological evaluation is needed. On ultrasound, axillary fibroadenoma presents as a hypoechoic, well-defined, benign-looking nodule [11].

Masses in axilla like ectopic breast tissue are clinically significant as they undergo physiological changes like in the normal breast tissue eg. pain and discomfort during pregnancy, lactation and menstruation [12]. This tissue may also undergo pathological changes and may pose a diagnostic challenge as it should be differentiated from lymphadenopathy, lipoma, phyllodes tumor, follicular cyst, hidradenitis, fibrocystic disease, intraductal papilloma, hamartoma or carcinoma [13,14] A study by Lee SR, retrospectively evaluated 39 patients with axillary fibroadenoma. Most patients presented with the complaint of an axillary lump and wanted to undergo evaluation due to the fear of malignancy. All the patients underwent excision via an axillary incision and were satisfied by the outcomes in terms of symptoms and cosmesis [15].

Due to the comparatively larger pool of differentials, there is often a delay in diagnosis of carcinoma arising from ectopic breast thereby leading to a poor prognosis [12]. Another aspect to keep in mind is that patients with ectopic breast tissue may have associated renal abnormalities. This has been explained in literature by the parallel development of the genitourinary system and the mammary structure, and is an important aspect to remember during evaluation of the patient [16].

## 4 Conclusion

Accessory breast tissue may undergo pathological changes and present as benign or malignant breast disease. These lesions pose a diagnostic dilemma due to the increased number of differential diagnoses and should be evaluated clinically, radiologically and by histopathology as for any other breast lesion. Furthermore, ectopic breast tissue should undergo routine annual screening as well.

## Ethical approval

Exemption was attained from the Ethical Review Committee.

## Sources of funding

This research received no funding or grant from any agency in the public, commercial, or not-for-profit sectors.

## Author contribution

Dr. Safna Naozer Virji: study concept, data collection, analysis and writing of the paper.

General surgery resident.

Aga Khan University Hospital, Karachi, Pakistan.

Dr. Lubna Mushtaque Vohra: study concept, data interpretation and writing of the paper.

Assistant Professor, Breast surgery.

Aga Khan University Hospital, Karachi, Pakistan.

Dr. Syeda Sakina Abidi: data interpretation, analysis and writing of the paper.

Fellow, Breast surgery.

Aga Khan University Hospital, Karachi, Pakistan.

Dr. Romana Idrees: data collection and interpretation.

Assistant Professor, Pathology and Laboratory Medicine.

Aga Khan University Hospital, Karachi, Pakistan.

## Registration of research studies

Not applicable.

## Guarantor

Dr. Safna Naozer Virji.

General surgery resident.

Aga Khan University Hospital, Karachi, Pakistan.

Dr. Lubna Mushtaque Vohra.

Assistant Professor, Breast surgery.

Aga Khan University Hospital, Karachi, Pakistan.

## Consent

Written informed consent was obtained from the patient for publication of this case report and accompanying images. A copy of the written consent is available for review by the Editor-in-Chief of this journal on request.

## Provenance and peer review

Not commissioned, externally peer-reviewed.

## Conflicts of interest

No conflict of interest.
